# Intriguing Carbon Flake Formation during Microwave‐Assisted Hydrothermal Carbonization of Sodium Lignosulfonate

**DOI:** 10.1002/gch2.201900111

**Published:** 2020-02-20

**Authors:** Giuseppe Melilli, Karin H. Adolfsson, Andrea Impagnatiello, Giancarlo Rizza, Minna Hakkarainen

**Affiliations:** ^1^ Department of Fibre and Polymer Technology KTH Royal Institute of Technology Teknikringen 58 SE‐100 44 Stockholm Sweden; ^2^ Laboratoire des Solides Iradiée Ecole Polytechnique Route de Saclay 91128 Palaiseau France

**Keywords:** carbon flakes, carbonization, hydrothermal process, lignosulfonate, microwaves

## Abstract

Elongated carbon structures, here denoted as carbon flakes (CF), are revealed after microwave‐assisted hydrothermal carbonization of sodium lignosulfonate. The morphology of formed CF is investigated by transmission electron microscopy and atomic force microscopy. Interestingly, a wide range of length distributions (between 100 and 700 nm) and a relatively constant aspect ratio and thickness are observed, indicating structures clearly different from the carbon spheres commonly formed during hydrothermal carbonization of lignocellulosic biomass. Moreover, X‐ray diffraction, Raman spectroscopy, Fourier transform infrared spectroscopy, and X‐ray photoelectron spectroscopy provide further information of the chemical structure, which consist mainly of nanographitic domains with a high degree of defects such as oxygenated functional groups, hybridized sp^3^ carbon, and aliphatic side chains. Furthermore, new insights into the formation mechanisms are uncovered and the formation is speculated to proceed through the combined effect of microwave irradiation and a heterogeneous solid–solid conversion. The formed CF are anticipated as highly interesting products for a variety of material applications.

## Graphical abstract

Formation of elongated carbon flakes is revealed after microwave‐assisted hydrothermal carbonization of sodium lignosulfonate. The formed carbon flakes are shown to consist mainly of nanographitic domains with a high degree of defects such as oxygenated functional groups. Furthermore, new insights into the formation mechanisms are uncovered. Carbon flakes are anticipated as highly interesting products for variety of material applications.

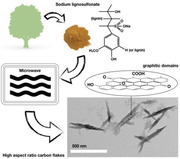

Biomass and biopolymers have caught a lot of attention as potential precursors for large variety of carbon‐based materials. There are different processes to convert biomass to high carbon content products, such as pyrolysis, gasification, hydrothermal carbonization (HTC), and flash carbonization.^1^ Low temperature HTC, performed below 300 °C, is a low energy consuming approach to produce oxidized hydrochar under relatively mild conditions.^2^, ^3^ Polysaccharides such as lignin, cellulose, chitosan, and starch are considered good candidates for production of high quality hydrochars.^4^, ^5^, ^6^ The carbonization mechanism of carbohydrates under HTC involves several steps: 1) hydrolysis/depolymerization of the molecules to the monomeric units, 2) (hydroxymethyl)furfural formation, 3) polymerization/condensation toward polyfurans, and 4) aromatization and intramolecular dehydration.^7^, ^8^, ^9^ The radial growth of the graphitic domains leads to the formation of spherical particles, named carbon spheres (CS). According to Sevilla et al.,^3^ the outer surface of CS contains higher concentration of reactive oxygen groups (hydroxyl, carbonyl, carboxylic, etc.), whereas the core has higher degree of aromatization.^9^ Moreover, the presence of polar oxygenated groups offers further possibilities for functionalization.^10^ The CS and functionalized CS have been used in wide range of applications such as drug delivery systems,^11^ antibacterial materials,^12^ CO_2_ sorbents, hydrogen storage,^13^ heavy metal,^14^, ^15^ and pharmaceutical adsorption.^16^

According to Kang et al.,^4^ the relatively low temperature during hydrothermal carbonization is not enough to completely dissolve lignin. The nondissolved lignin undergoes heterogeneous pyrolysis named solid–solid conversion. The main mechanisms involved in the solid–solid conversion consist of intramolecular rearrangement, dehydration, and decarboxylation reactions leading to the polyaromatic char formation as already reported for the hydrothermal carbonization of lignin^4^, ^17^ and corn stalk.^2^

The morphology, degree of carbonization, and porosity of the hydrochars can be modified using catalysts. Krishnan et al.^18^ reported the use of graphene oxide as a catalyst in hydrothermal carbonization of glucose and cellulose. The presence of graphene oxide (GO) sheets acted as nucleation and growth sites for seeding the carbonization product of glucose producing carbon platelets. Iron ions and iron oxide nanoparticles could effectively catalyze the hydrothermal carbonization of starch and rice grains under mild conditions (<200 °C). These catalysts also had significant influence on the formation of carbon nanomaterials with different shapes. In the presence of Fe‐ions, both hollow and massive carbon microspheres could be obtained. In contrast, the presence of iron oxide nanoparticles led to very fine, rope‐like carbon nanostructures.^19^ Tellurium nanowires were successfully utilized for synthesis of core–shell Te‐carbon‐rich composites with ultrathin and ultralong Te nanowires as the core component and carbonaceous matter with remarkable reactivity as the shell.^20^ Shi et al.^21^ reported that presence of minerals in biomass such as gumwood can act as catalytic sites for the formation of carbon nanotubes.

During series of work we have investigated the microwave‐assisted hydrothermal carbonization (MAHC) of different biomass resources.^5^, ^6^, ^11^, ^12^, ^39^ Here we revealed the formation of intriguing carbon flakes (CF) during MAHC of sodium lignosulfonate (SLS). New insights into morphology, chemical structure, and formation mechanisms were uncovered by combination of advanced microscopic and spectroscopic tools, including transmission electron microscopy (TEM), atomic force microscopy (AFM), X‐ray diffraction (XRD), Raman spectroscopy, Fourier transform infrared spectroscopy (FTIR), and X‐ray photoelectron spectroscopy (XPS).

CF were synthesized via microwave‐assisted carbonization reaction, similar to what was reported by Hassanzadeh et al.,^22^ for cellulose and waste paper. Briefly, 2 g of SLS was mixed with 20 mL of aqueous solution of 0.01 g mL^−1^ H_2_SO_4_ in a Milestone FlexiWAVE oven. The temperature was first set to increase to 240 °C under a ramp time of 20 min and then kept at isothermal conditions by input irradiation for 2 h (Power 1200 W). The resulting carbon product was filtered from the solution, washed with deionized water, and freeze dried for 2 d. Sulfuric acid was used to catalyze the decomposition of the soluble part of SLS. The effectiveness of sulfuric acid as a catalyst for the liquefaction of cellulose^7^ and hemicellulose decomposition of sugarcane bagasse^23^ has been reported previously.

AFM images of the carbonized products indicated the presence of irregular elongated structures (CF) with a wide range of length distributions between 100 and 700 nm. The thickness was measured by AFM (**Figure** **1**a) and it corresponded roughly to 5 nm. The TEM images (Figure 1b) further confirmed the presence of CF. Despite of the irregular shape, an estimation of the aspect ratio (length/width) based on the TEM images was calculated. The results showed that, in average, the length was five to six times larger than the lateral width which agrees with the elongated appearance. The selected area diffraction pattern (SAED) of CF (Figure 1c) showed clear diffraction spots characteristic for crystalline order. The sixfold symmetric diffraction points (labeled using the Miller‐bravais *hkil* notation) were consistent with the hexagonal lattice, which is typical for graphitic structures.^24^, ^25^, ^26^

**Figure 1:**
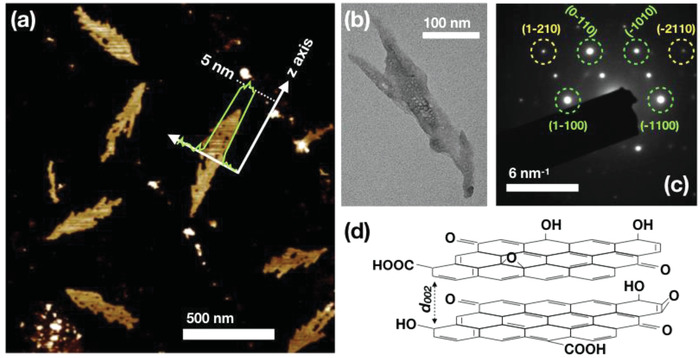
a) AFM image of CFs, b) TEM image of CF, c) SAED diffraction of a distorted hexagonal structure typical for a graphitic crystal, and d) schematic presentation of two superimposed oxygenated graphene layers derived by microwave‐assisted hydrothermal carbonization of SLS.

The XRD pattern (**Figure** **2**a) of CF exhibited two distinct peaks assigned to graphitic‐like structures: 2θ ≈ 24° and 43°, which correspond to the diffractions of (002) and (100) bands, respectively. Moreover, the gaussian deconvolution of the XRD pattern revealed a further peak named γ‐band, which has been reported for randomly oriented stacked lamellae of graphene in coals.^27^ The presence of this band is attributed to the presence of saturated structures such as aliphatic side chains attached to the edge of graphitic domains.^28^ It is well known that pristine graphite displays a typical peak attributed to the plane (002) at 2θ ≈ 26° (interlayer distance *d*_002_ = 3.36 Å).^20^, ^29^ The shift of (002) peak at lower 2θ in the pattern of CF graphite‐like structure corresponds to larger interplanar distance. According to the Bragg's law (2*d* sinθ = *nλ*), the *d*_002_‐spacing in CF, the average distance between two consecutive graphene planes, was calculated to be around 3.71 Å. The oxygen‐containing groups and hybridized sp^3^ carbon are a direct consequence of the microwave‐assisted hydrothermal carbonization of sodium lignosulfonate. The presence of these defects contributes to enlargening of the interlayer space in CF graphite‐like structures. The mean dimension of the crystallite (perpendicular to the plane of graphene) is calculated by applying the Sherrer equation (τ = *Kλ*/βcosθ), where *K* = 0.89 is the shape factor and β is the line broadening at half the maximum intensity, half width at half maximum (HWHM) expressed in rad. The resulting τ ≈ 11 Å corresponded to four stacked graphene layers. Moreover, a degree of aromaticity (*fa*) defined as the ratio between the carbon atoms of aliphatic chains and aromatics rings can be determined as: *A*_002_/*A*_002_ + *A*_γ_, where *A* is the integrated area under the corresponding peak.^30^ The *fa* for CF was found to be around 79%. A similar degree of aromaticity was reported by Zhang et al.,^31^ when Kraft lignin was thermally treated at 1000 °C. Considering the aforementioned example, the microwave assisted hydrothermal carbonization clearly provides high degree of aromaticity at relatively low reaction temperature (240 °C). One possible contributor could be higher local temperature at the surface of microwave absorbing carbonized products.^32^

**Figure 2:**
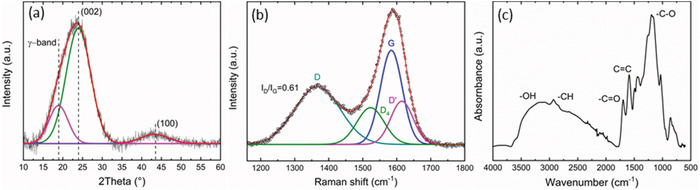
a) XRD pattern of CF, b) Raman spectrum of CF, and c) FTIR spectrum of CF.

The final structure of the CF was also investigated by Raman spectroscopy. The spectrum in Figure 2b confirms the presence of defects in the CF graphitic structure. In particular, the disorder‐induced band (D) can be easily recognized at 1367 cm^−1^. The D band is attributed to the scattering from defects which break the basic symmetry of the sp^2^ carbon in graphene sheet. The G‐band, instead, arises from planar configuration of sp^2^ bonded carbon in graphene. The G band for pristine graphite is located at 1580 cm^−1^ and appears as a narrow peak.^33^ Contrary to the pristine graphite, the Raman spectrum for the CF displays a broad G band at 1584 cm^−1^. The shift to higher frequency (respect to the pristine graphite) is a consequence of the defects such as bond‐angle disorder, bond‐length disorder, and sp^3^ hybridization,^34^ which reduce the long‐range order of the sp^2^ domains. The gaussian deconvolution of the D and G bands displays two additional bands named D_4_ and D'. The D' band partially merge with the G band^35^, ^36^ and it is located at 1615 cm^−1^. Similar to D band, D' is defect‐activated peak in the aromatic structure of graphitic domain.^37^ D_4_ band (≈1522 cm^−1^) indicates a change of hybridization in the graphene lattice in close proximity to the hydrogenated carbon atoms.^38^ The degree of ordering in CF can be estimated by the intensity ratio *I*_D_/*I*_G_, which corresponds to 0.61. Moreover, the ratio *I*_D_/*I*_G_ is inversely proportional to the in‐plane crystalline size of the sp^2^ domains (La).^39^ According to Cançado et al.,^40^ La is proportional to the fourth power of the laser wavelength λ (514.5 nm), which results in La (nm) = (2.4 × 10^−10^ × λ^4^) × (*I*_D_/*I*_G_)^−1^. The corresponding average size of the nanocrystalline domains is around 27 nm.

Several bands in the FTIR (Figure 2c) spectrum revealed the aromatization process achieved during the MAHC process. The aromatic rings are pointed out by the band at 1600 cm^−1^ which is associated to the stretching vibration C=C. The band around 800 cm^−1^ is also indicative of the aromatic structures and it is assigned to the C—H out‐of‐plane bending vibrations. Aliphatic structures can be deduced from the band at 3000–2815 cm^−1^ corresponding to the stretching vibrations of aliphatic C—H. Oxygenated functionalities were also identified. The broad peak between 3700 and 3000 cm^−1^ is associated with the stretching vibration of aliphatic O—H (hydroxyl and carboxyl). The C=O vibration at 1700 cm^−1^ corresponds to carbonyl while the peaks between 1200 and 1000 cm^−1^ correspond to C—O stretching vibrations from esters, phenols, and aliphatic alcohols and O—H bending vibrations.^3^, ^41^

X‐ray photoelectron spectroscopy was utilized to further characterize the chemical species present at the CF surface (**Figure** **3**). C 1s (Figure 3b) shows the major characteristic peaks attributed, respectively to aliphatic/aromatic carbon groups C—C/C=C/C—H*_x_* (285 eV), hydroxyl groups —C—OH (286.4 eV), carbonyl groups >C=O (287.6 eV) and carboxyl groups —COOH (289.3 eV).^3^, ^18^ CF also displays π–π* shake‐up satellite peak (291.4 eV) related to the extended delocalized electrons in aromatic ring.^42^ O 1s (Figure 3c) displays C—OH (533.4 eV) and C=O (531.9 eV). Interestingly, the sulfuric acid added as a catalyst during the MAHC process and the SLS sulfonic group were still found in the carbonized products. In particular, S 2p 3/2 indicates the presence of —S—H (164.2 eV) and —SO_3_—H (168.3, 169.3).^43^, ^44^

**Figure 3:**
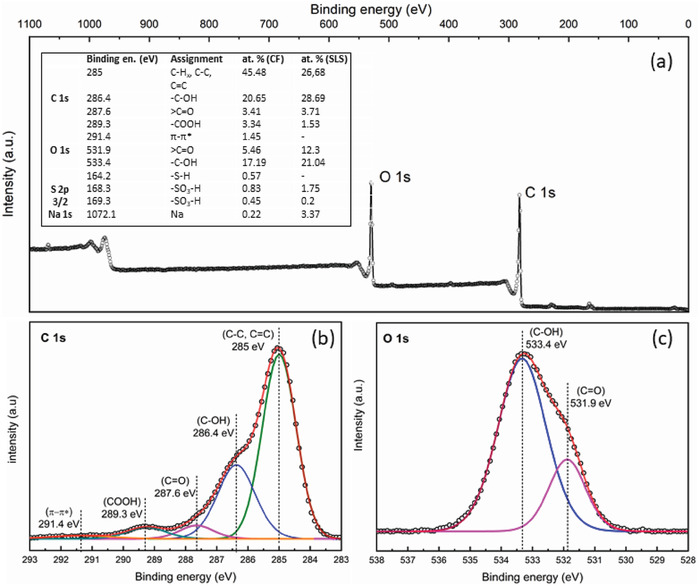
a) XPS survey spectra of CF. Inset: XPS assignment and at% of C 1s, O 1s, S 2p 3/2, and Na 1s. Narrow scan of b) C 1s and c) O 1s.

XPS shows the rich presence of oxygenated groups on the outer surface of the CF hydrochar. Based on the atomic percentages (inset in Figure 3a), the O/C ratio decreases after MAHC with respect to the SLS feedstock (from 0.55 to 0.30, respectively) as reported for similar lignocellulosic biomass conversions under similar conditions.^9^, ^45^ Based on these characterization results, the most probable mechanism for the CF formation is proposed in **Figure** **4**.

**Figure 4:**
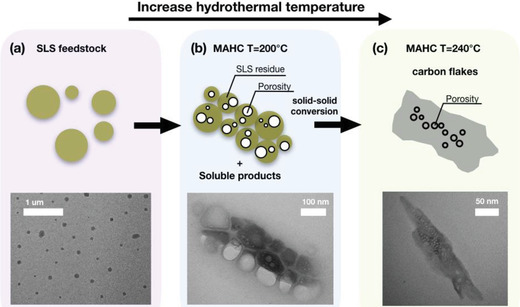
Scheme of CF formation: a) SLS feedstock, b) MAHC (*T* = 200 °C) resulting in self‐assembly of SLS residue, and c) MAHC (*T* = 240 °C) resulting in coalescence of carbonized product to form CF.

The SLS feedstock consisted mainly of spherical particles (TEM image, Figure 4a) with heterogeneous distribution of particle diameters (between 0.04 and few µm). After 2 h of MAHC treatment at *T* = 200 °C, the TEM image (Figure 4b) displayed the presence of nondissolved SLS fragments characterized by extensive porosity. Moreover, the SLS residues had high tendency toward self‐assembly promoted by microwave irradiation. In fact, microwaves can act as external electromagnetic field, which provides the driving force for the particle self‐assembly with a consequent reduction of the interfacial energy.^46^ This clearly indicates the preferential mechanism involved in the CF formation consisting of a solid–solid conversion which started at 200 °C and continued at higher temperature. As long as the graphitization proceeds the coupling between microwave and delocalized pi‐electrons becomes more intense providing a higher energy absorption.^17^, ^47^, ^48^ The local increase of the temperature promotes a further coalescence and aromatization of the carbonized products which results in the CF formation (Figure 4c).

We revealed the formation of elongated carbonaceous structures, here denoted as carbon flakes, during microwave‐assisted hydrothermal carbonization of SLS. The relatively large aspect ratio indicated structures different from the carbon spheres commonly formed during hydrothermal carbonization of lignocellulosic biomass. The CF morphology was in detail investigated by TEM and AFM. The CF length was measured to be between 100 and 700 nm, while the thickness was around 5 nm. XRD and Raman confirmed the presence of nanographitic domains with larger interlayer distance due to the presence of defects. The nature of the defects on the outer surface were investigated by FTIR and XPS which revealed the strong presence of oxygenated functionalities, hybridized sp^3^ carbon, and aliphatic side chains. We believe that the unusual CF morphology is the result of two combined effects: the heterogeneous solid–solid conversion which is a well‐known phenomenon in the hydrothermal carbonization of lignin biomass and the effect of microwave irradiation. Although at lower temperature the microwave irradiation contributed to the self‐assembly of the nonsolubilized SLS fragments, at higher temperatures the coupling between nanographitic domain and microwave irradiation led to the coalescence of the carbonized products resulting in the final CF structure.

## Conflict of Interest

The authors declare no conflict of interest.
